# Immune Profiling and Precision Medicine in Systemic Lupus Erythematosus

**DOI:** 10.3390/cells8020140

**Published:** 2019-02-10

**Authors:** Yasuo Nagafuchi, Hirofumi Shoda, Keishi Fujio

**Affiliations:** Department of Allergy and Rheumatology, Graduate School of Medicine, The University of Tokyo, 113-8655 Tokyo, Japan; shoda-tky@umin.ac.jp (H.S.); kfujio-tky@umin.ac.jp (K.F.)

**Keywords:** systemic lupus erythematosus, immune profiling, single-cell analysis, transcriptome, precision medicine

## Abstract

Systemic lupus erythematosus (SLE) is an autoimmune disorder with a wide range of clinical symptoms. Enormous progress has been made in the immunological and genetic understanding of SLE. However, the biology of disease heterogeneity in SLE has remained largely unexplored. Human immune profiling studies, helped by recent technological advances especially in single-cell and “omics” analyses, are now shedding light on the cellular and molecular basis of clinical symptoms and disease flares in individual patients. Peripheral blood immunophenotyping analysis with flow cytometry or mass cytometry are identifying responsible cell subsets and markers characteristic of disease heterogeneity. Transcriptome analysis is discovering molecular networks responsible for disease activity, disease subtype and future relapse. In this review, we summarize recent advances in the immune profiling analysis of SLE patients and discuss how they will be used for future precision medicine.

## 1. Introduction

Systemic lupus erythematosus (SLE) is a chronic autoimmune disease characterized by a broad spectrum of clinical symptoms and arrays of autoantibodies, such as antinuclear antibodies (ANA) and anti-double strand DNA (dsDNA) antibodies. Both genetic and environmental factors contribute to the onset of SLE. Concordance rates of 20 to 30% in monozygotic twins imply the importance of environmental factors in genetically predisposed individuals [1]. SLE primarily affects women of childbearing age. The female-to-male ratio of about 9:1 suggest a role of female sex hormones [2]. Exposure to ultraviolet light exacerbates skin disease in SLE and can trigger disease onset by induction of apoptosis in the skin [3]. Other environmental factors including cigarette smoking, chemicals, drugs, viral infections and gut microbiota [4] can also act as environmental triggers for SLE.

In the pathophysiology of SLE, there is an imbalance between cellular apoptosis and disposal of apoptotic material [5]. During the course of apoptosis, nuclear antigens and nucleic acids become accessible to the immune system. Persistent apoptotic debris can stimulate an inflammatory response through the activation of nucleic acid recognition receptors, such as members of the Toll-like receptor (TLR) family. Nucleic acid recognition receptors are strongly associated with type I interferon (IFN) production. Type I IFNs and other cytokines promote B-cell differentiation and loss of tolerance. B cells respond to nucleic acids or nuclear antigens and produce autoantibodies against them. Tolerance checkpoints can be bypassed when B cells are activated by TLR9 [6] or BLyS/ BAFF, a cytokine promoting B-cell survival and development [7,8].

In the 1950s, corticosteroids were introduced for treatment of SLE, and the 5-year survival of SLE in developed countries gradually increased to >95%, plateauing in the 1990s [9]. Major causes of death in SLE patients shifted from lupus activity to infection or cardiovascular events that can be related to treatment [10]. In most patients, active disease is controlled by corticosteroid treatment combined with immunosuppressant drugs such as cyclophosphamide introduced in the 1970s, with mycophenolate mofetil becoming available in 2000s. Treatment guidelines or recommendations are available from academic societies around the world [11,12,13,14]. Treat-to-target has been proposed, with a target of clinical remission or low disease activity that predicts favourable long-term outcomes [15]. However, controlling disease activity life-long without or with use of a minimal amount of immunosuppressants is a clinical challenge. The goal of prolonged remission or low disease activity is achieved only in a small proportion of patients [16,17]. Unpredictable and frequent relapses of active SLE, flares, and difficulties in maintaining a state of remission have persuades patients and physicians to continue immunosuppressive treatment (with accompanying side effects) to control disease activity.

In addition to disease activity, SLE patients are stratified and treated according to organ involvement, such as the kidney. The pattern of autoantibodies is also correlated to some SLE phenotypes [18]. For example, anti-dsDNA antibodies are associated with lupus nephritis [19,20]. However, these clinical stratifications do not necessarily reflect the biological heterogeneity of SLE.

Belimumab, a monoclonal antibody against BLyS/ BAFF was approved as the first targeted biological therapy for SLE in 2011 [21,22,23]. At least 74 targeted treatments utilizing IFN, cytokines, chemokines, and B-cells are under clinical trial for SLE [24]. With increasing treatment options, identification of biomarkers that stratify SLE patients to adequate treatment is an urgent need.

**Figure 1:**
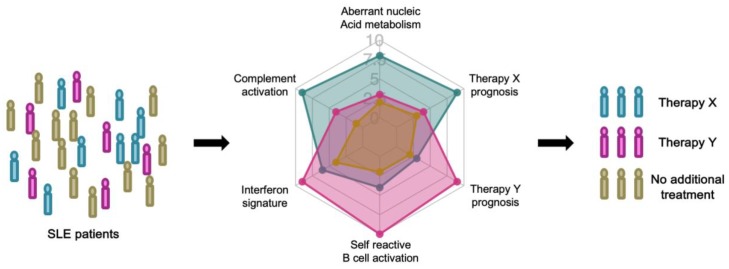
Stratified treatment of systemic lupus erythematosus (SLE) patients based on prognostic and predictive biomarkers.

A biomarker is a characteristic that can be objectively measured as an indicator of a normal or pathological biological processes, or as an indicator of response to therapy [25]. Immune profiling, or immune cell profiling, realized by technological advances in multi-dimensional analysis of immune cells, will enable deeper understanding of disease pathogenesis and heterogeneity and the discovery of novel biomarkers [26]. Prognostic biomarkers to forecast the natural course of the patient or predictive biomarkers to forecast the response to therapy are necessary for precisely tailored treatment, precision medicine, of SLE (Figure 1) [27].

In the coming era of precision medicine, SLE patients will be stratified by immune profiling. Each patient will be longitudinally evaluated with prognostic biomarkers that predict the natural course of the disease. Prognostic biomarkers reflect immunological characteristics, such as a high interferon signature. When disease relapse is predicted with high confidence, the patient’s treatment can be guided by predictive biomarkers that project the response to each therapy.

## 2. Genetics of SLE and Immunological Insights

### 2.1. Monogenic SLE

Identification of causal mutations in rare monogenic lupus families or lupus-like autoimmune syndrome gave crucial insights into the pathogenesis of SLE. Lupus-prone families with primary defects of classical complement pathways (C1q, C1r/s, C2, C4A and C4B) revealed the importance of this pathway for lupus pathogenesis [28]. Complement is essential for opsonization and clearance of immune complexes and apoptotic cells. In addition, gene mutations involved in DNA processing during apoptosis cause lupus-like systemic autoimmunity [29]. Pediatric brain disease with a lupus-like symptom, Aicardi-Goutieres syndrome (AGS) is a prototypic example. AGS involves mutations in RNases (*RNASEH2A*, *RNASEH2B*, *RNASEH2C*), a DNase (*TREX1*), double-stranded RNA (dsRNA) editing (*ADAR*), dsRNA recognition and binding (*IFIH1*), and activation of the innate immune system (*SAMHD1*, *IFIH1*) [30]. Twelve of 20 AGS patients presented SLE-like characteristics, such as antinuclear antibodies (ANA), skin disease, thrombocytopenia, leukocytopenia, and arthritis [31]. AGS is now considered as a ‘type I interferonopathy’ because type I IFN production is constitutively upregulated in AGS patients and is directly relevant to pathogenesis [32]. Decreased DNase 1 activity by heterozygous nonsense *DNASE1* mutation has been reported in 2 of 20 SLE patients [33]. Additionally, *DNASE1L3* mutation causes familial SLE with positive ANA, anti-double strand DNA (dsDNA) antibodies, and anti-neutrophil cytoplasmic antibodies (ANCA) and low C3/C4 [34].

A homozygous missense mutation in *PRKCD*, encoding protein kinase δ (PKCδ) was reported in a family of juvenile-onset SLE [35]. PKCδ is a serine/threonine kinase that has been implicated in the negative selection of self-reactive B cells and control of cell proliferation in mice [36,37]. The missense mutation of *PRKCD* in affected families resulted in reduced activity of PKCδ, leading to resistance to apoptosis and increased B-cell proliferation.

These insights from Mendelian SLE or related syndrome families highlight the central role of nucleic acid metabolism, the complement pathway, and self-reactive B cells in human SLE pathogenesis.

### 2.2. Polygenic SLE

A genome-wide association study (GWAS) consists of hypothesis-free screening for linkage between loci and common multifactorial diseases, such as SLE. The association between GWAS-identified common single nucleotide polymorphisms (SNPs) and targeted traits is statistically robust. However, the effect size of most individual loci is small, as shown by typical odds ratios of identified loci ranging from 1.1 to 1.5 in large scale GWASs. Most of the identified SNPs lie in noncoding regions and affect gene expression through transcriptional or epigenetic modifications.

More than 100 loci have been shown to be robustly associated to SLE, especially in European and/or Asians GWASs [38,39]. Some of the reported genes are related to aberrant recognition of self-nucleic acid (*NCF1*, *NCF2*, *FCGR2A*, *ITGAM* and others), type I IFN overproduction/TLR signaling (*IFIH1*, *IRF5*, *TNFAIP3* and others) and defective immune cell signaling (*BLK*, *TNFSF13B* and others). In other cases, the immunological function is unknown.

The human leukocyte antigen (HLA) region encodes more than 120 functional genes, such as HLA molecules involved in antigen presentation, complement components C2 and C4 and cytokine TNF-α [38,39]. Most of the genes in this region are immune-related and have a strong linkage equilibrium. HLA-DR and -DQ loci are consistently associated with SLE in different ethnic populations. The involvement of non-HLA class III region genes has also been strongly supported by GWAS results [40,41,42].

Expression quantitative trait loci (eQTL) analysis links each locus to variations of gene expression in each cell or tissue type. eQTL analysis of GWAS-identified loci with cell type-specific regulation of disease loci, such as *BLK* in B cells and *JAZF1* in T cells [40]. SLE GWAS SNPs are enriched for B cell- and T-cell-specific gene expression and epigenetic enhancer marks [41,43]. Genetic risk score calculated by adding cumulative SLE-associated risk alleles weighted by SLE risk odds ratios revealed a higher genetic risk in non-European than European individuals, which may help explain the increased prevalence of SLE in non-Europeans [44].

SLE is a clinically heterogeneous disease and some phenotype-related loci have been reported, such as *PDGRFA* in lupus nephritis and *ITGAM* in arthritis [45,46]. However, the genetic architecture of subphenotypes of SLE is not fully elucidated. Reanalysis of existing GWAS with clinical subphenotypes may identify novel loci in association.

## 3. Immune Profiling of SLE

SLE is an autoimmune disease mediated by both innate and adaptive immune systems. Therefore, profiling of immune cells is a promising approach for biomarker discovery. Immunological memory enables immune systems to specifically and efficiently recognize antigens that they encountered, sometimes for a lifetime. Memory T cells and B cells that are long-lived and specific for particular antigens are the classical cells responsible for immune memory [47,48]. Defects in immune tolerance cause this efficient immune system to provoke autoimmunity that typically lasts a lifetime. Profiling of immune cells can reflect its history (for example, by analyzing autoantibody repertoire). Additionally, immune profiling can reflect the current status of an immunological system related to disease activity and future responses to treatment (for example, by analyzing IFN signature or cell subset frequencies).

Several methods have been developed to identify human immune cells. Flow cytometry or mass cytometry, targeting pre-specified marker proteins, allows quantification of immune cell composition at single-cell resolution. Transcriptome analysis, by microarray or RNA-sequencing (RNA-seq), allows genome-wide messenger RNA expression level quantification, methods that are fruitful for identifying pathways or modules of genes that are related to disease activity or prognosis. Proteome analysis can profile every protein or targeted proteins, such as autoantigens, thus reflecting immune system activation in SLE.

### 3.1. Flow Cytometry

Flow cytometry is a popular and powerful tool to characterize immune cell populations and functions. It utilizes fluorescent markers to label cells, and each cell is subjected to laser illumination to characterize expressed antigens/fluorochromes. Today, multicolor flow cytometry can simultaneously analyze more than 10 protein markers and a maximum of 50 markers on a single cell. Cell membrane permeabilization enables detection of not only cell surface markers, but also of cytokines and transcription factors in the cytoplasm and nucleus.

Kubo et al. performed flow cytometric phenotyping of circulating T cells, B cells and dendritic cell from 143 SLE patients and classified the patients into three clusters [49]. Patients with a high percentage of follicular helper T cells (Tfh) were more resistant to treatment with cyclophosphamide, mycophenolate mofetil, or calcineurin inhibitors, in addition to high-dose glucocorticoids. Tfh cells are essential for B-cell maturation and autoantibody production [50]. The proportions of CXCR5^+^ CCR7^low^ PD-1^high^ Tfh cells or CXCR5^high^ ICOS^high^ PD-1^high^ Tfh cells were also reported to be associated with disease activity in SLE [51,52]. OX40 ligand on myeloid antigen-presenting cells, induced by immune complexes containing RNA, is reportedly involved in the mechanism promoting Tfh responses in SLE [53].

Rituximab, an anti-CD20 monoclonal antibody targeting B cells, is a treatment option for severe lupus. However, randomized control trials did not confirm the efficacy [54,55]. Yusof et al. reported that B-cell depletion at 6 weeks predicts clinical response to rituximab treatment in SLE with an odds ratio of 3.22 in multivariate analysis [56]. Monitoring of B-cell depletion and individually tailored rituximab treatment was also tried in ANCA-associated vasculitis and resulted in fewer rituximab infusions with similar clinical efficacy [57]. Measuring disease-relevant cell population frequency is a candidate approach to the monitoring of immune system status or to predict treatment response.

Additional immune cell subpopulations are gaining attention in the pathophysiology of SLE. Low-density granulocytes constitute a distinct pro-inflammatory neutrophil subset found in SLE, and they exhibit enhanced spontaneous NETosis [58]. SLE low-density granulocytes release pro-inflammatory oxidized mitochondrial DNA in a mitochondrial reactive oxygen species- (ROS) dependent manner [59]. NETs opsonized by autoantibodies can stimulate plasmacytoid dendritic cells (pDCs) to synthesize IFN-α. In healthy individuals, pDCs drive the differentiation of CD19^+^ CD24^hi^ CD38^hi^ immature B cells into IL-10-producing CD24^+^CD38^hi^ regulatory B (Breg) cells and plasmablasts by the release of IFN-α and CD40 engagement [60]. In SLE, pDCs fail to induce Breg cells because of the high IFN-α concentration. CXCR5^−^ CXCR3^+^ PD1^hi^ CD4^+^ helper T cells (Th10 cells), distinct from Tfh cells, help B cells through interleukin-10 and succinate and are expanded in SLE blood and kidneys [61]. A subset of CD19^+^ IgD^+^ CD27^−^ naïve B cells persist in the circulation for months and differentiate into autoantibody secreting cells [62]. Additionally, autoreactive CD27^−^ IgD^−^ CXCR5^−^ CD11c^+^ (DN2) B cells were recently shown to be disease-relevant in SLE patients [63]. DN2 cells, characterized by high transcription factor T-bet expression, are derived from naïve B cells and generate autoreactive plasma cells likely through extrafollicular differentiation. They show similarities to previously reported age- or autoimmune-associated B cells (ABCs) [64,65].

Diamond et al. developed a flow cytometric assay to identify ANA-reacting B-cells using biotinylated nuclear extracts [66,67]. Malkiel et al. showed an impairment of anergy induction in ANA^+^ naïve B cells in SLE patients, assessed by the percentage of IgM^low^ ANA^+^ naïve B cells, and restoration with belimumab treatment [66]. Suurmond et al. observed increased numbers of ANA^+^ IgG^+^ plasma cells in SLE patients, as well as in lupus-prone MRL/lpr and NZB/W mice. This increase was suggested to be the result of aberrant IgG+ plasma cell expansion, not by impaired antigen-specific tolerance checkpoints [67]. While the clinical utility of ANA+ B-cell analysis has not been assessed, profiling of autoantibody-specific B-cells is an attractive approach to monitoring B-cell immunity in SLE.

### 3.2. Mass Cytometry

As an alternative to flow cytometry, mass cytometry, also known as Cytometry by time-of-flight (CyTOF), uses heavy metal isotopes to label antibodies, and the labeled cells are analyzed by high-throughput spectrometry on a single-cell level. Typically, it analyzes >40 markers simultaneously and significantly augments the ability to evaluate complex cellular systems in high dimension. In flow cytometry, fluorophore emission spectra overlap, which makes it difficult to resolve the colors in multicolor analysis. This feature limits the number of measurable parameters and “compensation” is needed to account for spillover of light among them. In contrast, mass cytometry is able to discriminate isotopes of different atomic weights with high accuracy and the need for compensation is reduced [68]. However, it cannot recover living cells after analysis because cells are atomized and ionized for analysis.

Application of this new technology to leukemia and carcinoma has successfully identified potential biomarkers to forecast prognosis [69,70,71]. Mass cytometry is also being used in studies of rheumatology. Mass cytometric analysis of peripheral blood or synovial cells of rheumatoid arthritis patients identified candidate disease-associated T-cell populations, PD-1^hi^ CXCR5^−^ peripheral helper T cells and CD27^−^ HLA-DR^+^ effector memory T cells [72,73].

Using mass cytometry, O’Gorman et al. performed systematic ex vivo Toll-like receptor activation analysis of SLE [74]. Among various immune cell subsets analyzed, CD14^hi^ monocytes exhibited the most polyfunctional cytokine expression patterns, with more than 80 distinct cytokine combinations. Eight newly diagnosed untreated SLE patients shared a distinct monocytic chemokine signature compared to healthy volunteers. They also analyzed 10 newly diagnosed and untreated pediatric SLE patients with mass cytometry and identified a distinct monocyte signature characterized by MCP1, Mip1β and IL-1RA [75]. The cytokine signature was induced in monocytes from healthy volunteers by adding plasma from clinically active SLE patients and was abrogated by selective Janus kinase (JAK) inhibition. This signature was partially abrogated by interferon-α/β receptor (IFNAR) blockade, suggesting a role of IFN pathway for this monocyte signature. These promising results show the utility of mass cytometry in unveiling the pathophysiology of SLE. Larger sample size analysis is needed to dissect disease heterogeneity and discover prognostic and predictive biomarkers in SLE.

### 3.3. Microarray and RNA-seq

Initially, microarrays were used to study the transcriptome, the full range of messenger RNAs (mRNAs), or all RNAs in various cell populations [76]. It utilizes hybridization between fluorescently labelled complementary DNA (cDNA) with custom-made microarrays or commercial high-density oligo microarrays. They are relatively inexpensive, and they rely on known genomic sequences. RNA-seq directly determines cDNA sequences with next generation sequencers. RNA-seq offers several advantages over microarrays. RNA-seq is not dependent on prior knowledge of genomic sequences, and the dynamic range to quantify gene expression level is superior to microarrays. It can also be applied to analyze different isoforms and allele-specific expression [77]. RNA-seq is expected to substitute for microarrays in the near future. Most of the existing SLE transcriptome data are based upon microarray analysis.

Early microarray analysis led to the development of gene expression signatures, such as the IFN signature, i.e., the group of transcripts modified by IFN exposure. In 2003, two groups almost simultaneously identified IFN-induced genes by microarray comparisons of peripheral blood mononuclear cells (PBMC) in active SLE [78,79]. The expression of IFN signature genes was correlated to disease activity, as confirmed by later cross-sectional analyses [80,81,82]. An IFN signature is inducible by type I IFNs (IFNα or IFNβ). Anifrolumab, a type I IFN receptor antagonist, showed significant, although not strong, decreases of disease activity in moderate to severe SLE in a phase II clinical trial [83]. Anifrolumab showed a greater effect in patients with a high IFN signature. This clinical efficacy confirmed the role of IFN signaling as a disease-relevant pathway of SLE. The importance of this pathway is also noted in other autoimmune diseases, such as rheumatoid arthritis, Sjogren syndrome and systemic sclerosis [84,85,86].

Chaussabel et al. applied modular analysis that identified sets of coordinately expressed transcripts to PBMC microarray data from 239 individuals and found two SLE disease activity-related transcriptional modules, IFN-inducible and neutrophil genes [87]. Their analysis also revealed complex IFN signatures in SLE that are composed of three modules and involve both IFNα signature and also IFNβ and IFNγ [88]. Recently, a two-score system for IFN status has also been proposed, based on factor analysis of 31 IFN-stimulated genes [89,90]. Large scale IFN signaling network analysis (by the Immunological Genome Project Consortium) showed five regulatory modules of IFN signaling [91]. Most SLE-associated genes were from one cluster and appeared to require TYK2. These genes were sensitive to TYK2 deletion in mice. TYK2 is a JAK kinase activated by IFN binding to the IFNAR receptor and one of the GWAS-identified locus for SLE [38].

Banchereau et al. transcriptionally profiled 156 pediatric SLE patients longitudinally [92]. With 924 whole blood samples analyzed with microarrays, the plasmablast signature was the most robust disease activity biomarker in their analysis. While IFN response and plasmablast signature were involved in all patients, the neutrophil module was only activated in patients with active nephritis. They proposed a model of gradual disease progression, with early increases in IFN response and differentiation of B cells into plasmablasts, and late kidney disease and full-blown systemic inflammation fueled by myeloid cells, including neutrophils. They stratified SLE patients into seven clusters based on personalized immune-monitoring correlates of disease activity. Interestingly, the disease activity-related correlates differed from cluster to cluster, showing high molecular heterogeneity in SLE patients.

One limitation of whole blood transcriptome analysis is the fact that the differences in transcriptomes are largely influenced by the composition of immune cells in each sample. For example, a plasmablast signature can reflect increased frequency of plasmablasts and it is difficult to assess the qualitative difference in B-cell differentiation. To address qualitative changes in each cell subset, analysis of purified populations is needed. Lyons et al. isolated CD4 and CD8 T cells, B cells, monocytes and neutrophils from SLE and vasculitis patients and performed microarrays together with whole PBMC analysis. A substantial number of differentially expressed genes was only identified with purified cells and discrimination between patient groups was improved with purified monocytes [93]. McKinney et al. analyzed microarrays of purified CD8 and CD4 T cells with network and module analysis. They found that CD8 T-cell exhaustion negatively correlated with CD4 T-cell co-stimulation and that it indicated a better prognosis in SLE and in ANCA-associated vasculitis patients [94,95]. A candidate prognosis marker in the transcriptome of unseparated PBMCs, *KAT2B*, was also identified in their analysis. Their study emphasizes the utility of transcriptomic analysis of purified cell subsets.

To guide the initial therapy of active lupus nephritis, kidney biopsy and histological evaluation is the gold-standard [96]. Parikh et al. analyzed the expression of 511 immune-response genes with NanoString in kidney biopsies from 19 patients [97]. Genes responsible for clinical responses included IFN pathway genes, while complement genes were mainly found in non-responders. Transcriptome profiling of biopsy specimens may provide additional biomarkers for clinical histology, although access to affected organs, such as kidney, is more limited than blood sampling.

Recently, several groups in this field have shown the utility of RNA-seq. Shi et al. performed RNA-seq of both coding and noncoding RNAs of monocytes from 9 SLE patients [98]. They found significantly altered splicing patterns and novel transcripts. Rai et al. compared peripheral blood RNA-seq data from 28 SLE patients [99]. Multiple cytokine signaling pathways were specifically dysregulated in anti-dsDNA+ patients, whereas IFN signaling was predominantly dysregulated in anti-extractable nuclear antigen (ENA)+ patients. Zhang et al. performed RNA-seq in combination with H3K4me3 Chip-seq in T cells, B cells and monocytes from 6 SLE patients [100]. Although their analysis was limited by the small number of participants, it showed both shared and cell-specific changes in gene expression and epigenetics.

### 3.4. Single-Cell Expression Profiles and RNA-seq

Application of RNA-seq to single cells (single-cell RNA-seq) allows comprehensive analysis of immune cells at single-cell resolution [101,102]. In combination with plate-based or droplet-based single-cell isolation, hundreds, thousands, and more cells can be simultaneously analyzed for their transcriptome. It can be used to identify and characterize disease related immune cell subsets. The transcriptional signatures of these immune cells enable the identification of novel biomarkers. Additionally, immune repertoire analysis of single cells can identify T-cell or B-cell receptor pairs.

Jin et al. analyzed single-cell gene expression profiles using a monocyte-related transcription panel to assess SLE patient monocytes [103]. Unsupervised hierarchical clustering of SLE monocytes demonstrated that independent clusters of cells were related to disease activity, type I IFN and medication use. Accelerating Medicines Partnership (AMP) in Rheumatoid Arthritis and Lupus Network is a public-private partnership created to develop new ways of identifying and validating promising biological targets for diagnostics and drug development. In an early report from the AMP project, Der et al. applied single-cell RNA-seq to kidney and skin biopsy samples from 16 lupus nephritis patients [81]. Keratinocytes from the skin of those patients also revealed upregulation of IFN-inducible genes. With greater sample sizes, their analysis may help to identify disease-relevant or disease subtype-related populations that only exist in affected organs.

### 3.5. Autoantibody Repertoire and Proteomics

More than 180 autoantibodies have been found in the blood of SLE patients and different patients may exhibit different autoantibody profiles [104]. Autoantigen arrays are a high-throughput autoantibody screening platform based on antigen-antibody reactions. The arrays are produced by immobilizing hundreds or more diverse autoantigens on the coated surface of glass slides. The arrays are reacted with diluted samples, such as serum, and the autoantibodies bound to their corresponding antigens are detected with the fluorophore-conjugated second antibodies. Li et al. constructed protein microarray bearing about 30 antigens known to be expressed in the kidney and identified five distinct clusters of IgG autoreactivity in the sera of lupus patients, and two of the clusters showed association with disease activity [105]. Huang et al. used protein arrays containing over 5000 recombinant human proteins to profile the autoantibodies in the sera of SLE patients. Four novel antigens including CLIC2, were identified as potential targets of autoantibodies in SLE [106]. ELISA experiments confirmed the presence of autoantibody to CLIC2 in 28% of SLE patients. Kinloch et al. screened cloned activated B cells isolated from renal biopsy specimens of lupus nephritis patients. They found that vimentin was a dominant autoantigen targeted in lupus tubulointerstitial nephritis [107]. These results show that protein arrays can contribute to the discovery of novel autoantibodies. Detection of certain patterns of autoantibodies may help to forecast disease activity or response to therapy.

Autoantibodies, such as anti-dsDNA antibodies and anti-Ro antibodies, are present many years before the diagnosis of SLE and progressively accumulate until clinical onset [108]. Recent analysis with multiplex bead-based antigen detection for 398 antigens, showed that the number of epitopes recognized by autoantibodies generally do not change over six years in established SLE patients [109]. dsDNA and histone H3 autoantibodies were notable exceptions and constituted disease activity markers. An increase in antibodies to the U1-RNP epitopes at the time of new organ involvement was noted, suggesting intramolecular epitope spreading may be more sensitive to disease activity.

Of note, autoantigen microarrays and bead-based multiplex analysis are both “biased” proteomic analyses to the extent that they target only known peptides [110]. Typical “unbiased” proteomic analysis with mass spectrometry first separates proteins using gel-based techniques and identifies proteins with mass spectrometry [111]. More than 241 SLE candidate proteomic biomarkers have been discovered with mass spectrometry analysis and 28 candidate biomarkers were validated in independent cohorts or studies. Ferreira et al. identified candidate lupus diagnosis and activity markers with two-dimensional differential gel electrophoresis and mass spectrometry [112]. Limited numbers of lupus participants, typically 5 to 20, and variable experimental conditions in each study warrant validation of these candidate protein biomarkers.

## 4. Toward Precision Medicine in SLE

At present, no validated biological biomarker exists to predict disease course and treatment response with high reliability and reproducibility [113]. Conventional disease assessment methods, including the use of acute phase markers, such as erythrocyte sedimentation rate (ESR) and C-reactive protein (CRP), and anti-dsDNA antibodies are of limited sensitivity and specificity.

Future enhancements of prognostic and predictive biomarkers will improve the assessment and clinical management of SLE (Figure 1). It will help the diagnosis, evaluation of disease activity and therapeutic decisions. Precise biological evaluation of disease activity by use of prognostic biomarkers will quantitatively evaluate the possibility of future relapse or organ damage. In that way, clinical decisions regarding intensification or tapering of treatment can be improved. Predictive biomarkers will forecast the clinical response and possible adverse reactions and will aid clinical decision making based upon the available treatment options.

Transcriptome analysis of whole blood or targeted immune cells is a promising way to identify gene expression biomarkers. There have been recent successes in discovering gene modules closely related to disease activity, disease subtype, or future relapse [92,94,95]. The bulk transcriptome reflects the average expression of individual cells. A small cell fraction may be responsible for the transcriptome difference. For example, a CD8 T-cell exhaustion signature can be the result of an expanded small fraction of exhausted CD8 T cells. To fully characterize a disease-relevant small cell subset that is responsible for transcriptome change, deeper immune phenotyping, probably by mass cytometry or single-cell RNA-seq in combination with bulk transcriptome analysis is needed. Additionally, quantifying the expression of a small number of mRNAs, as is done in IFN signature assays [89,90], may serve as an alternative approach to monitor transcriptome signatures.

Reproducibility is one of the most important issues for clinical application of biomarkers to precision medicine. Thus, candidate biomarkers must be assessed with a standardized protocol. For example, standardization of immune cell phenotyping has been proposed [114]. However, researchers continue to identify new candidate immune cell populations that drive SLE including Th10 cells and DN2/ABCs described above. These examples of the diversity of candidate biomarker populations provoke a dilemma between standardized and exploratory research protocols. Exploratory immune profiling “omics” analysis needs to be followed by standardized multi-center validation studies, targeting a small number of candidate biomarkers. Identification of reliable and reproducible biomarkers is the key to realize precision or personalized medicine in SLE.

## 5. Conclusions

The heterogeneous nature of SLE is being elucidated by human immune profiling studies. Disease-relevant immune subsets identified by immunophenotyping analysis, or gene network signatures identified by transcriptome analysis, if validated by independent study, may serve as novel biomarkers to realize future precision medicine in SLE. Prognostic biomarkers will reflect immunological abnormalities in each patient and will forecast their natural course. Importantly, predictive biomarkers will forecast the response to treatment options. These biomarkers will improve and guide future clinical management of SLE.
